# A quantitative philology of introspection

**DOI:** 10.3389/fnint.2012.00080

**Published:** 2012-09-24

**Authors:** Carlos G. Diuk, D. Fernandez Slezak, I. Raskovsky, M. Sigman, G. A. Cecchi

**Affiliations:** ^1^Department of Psychology, Princeton Neuroscience Institute, Princeton UniversityPrinceton, NJ, USA; ^2^Department of Computer Science, University of Buenos AiresBuenos Aires, Argentina; ^3^Department of Physics, University of Buenos AiresBuenos Aires, Argentina; ^4^Computational Biology Center, T.J. Watson IBM Research CenterYorktown Heights, NY, USA

**Keywords:** introspection, latent semantic analysis, neuroscience, Google n-grams, semantic cognition

## Abstract

The cultural evolution of introspective thought has been recognized to undergo a drastic change during the middle of the first millennium BC. This period, known as the “Axial Age,” saw the birth of religions and philosophies still alive in modern culture, as well as the transition from orality to literacy—which led to the hypothesis of a link between introspection and literacy. Here we set out to examine the evolution of introspection in the Axial Age, studying the cultural record of the Greco-Roman and Judeo-Christian literary traditions. Using a statistical measure of semantic similarity, we identify a single “arrow of time” in the Old and New Testaments of the Bible, and a more complex non-monotonic dynamics in the Greco-Roman tradition reflecting the rise and fall of the respective societies. A comparable analysis of the twentieth century cultural record shows a steady increase in the incidence of introspective topics, punctuated by abrupt declines during and preceding the First and Second World Wars. Our results show that (a) it is possible to devise a consistent metric to quantify the history of a high-level concept such as introspection, cementing the path for a new quantitative philology and (b) to the extent that it is captured in the cultural record, the increased ability of human thought for self-reflection that the Axial Age brought about is still heavily determined by societal contingencies beyond the orality-literacy nexus.

## Introduction

The period in human history ranging from 800 BCE to 200 BCE marked a radical transformation in world civilizations, in particular those that constitute the Western tradition. Famously termed the Axial Age by Jaspers (1953), this period produced world religions and philosophies that are still pillars of modern culture. Many scholars have argued that one of the most prominent consequences of this transition was a change in consciousness, from a “homeostatic”, living-in-the-present form, to a self-reflective and time-expanding inner life (Schwartz, 1975; Eisenstadt, 1986; Armstrong, 2006). Studying two foundational texts of Western civilization, the Bible and the Homeric saga, Julian Jaynes (2000) argued that the change in conscious life that took place during the Axial Age is part of a larger introspection-increasing arc explicitly expressed in the textual narrative. The most cursory analysis of the Bible shows how the wrathful, interventionist god of the Old Testament, who expels Adam and Eve from the Garden of Eden (Genesis 6:22), is followed by the rich inner life of the New Testament, where Jesus asks the accusers of an adulterous woman to examine their own guilt (John 8:1). The striking differences between the Iliad and the Oddyssey in the way the characters' behaviors are attributed, respectively, to divine intervention or to the individual's volition, have been pointed out in numerous studies (Dodds, 1951; Adkins, 1970; Onians, 1988; De Jong and Sullivan, 1994). The impulsive, irreflexive heroes of the Iliad, driven by passions insufflated into them by the gods, give way to the wily, cunning Odysseus who outsmarts Polifemo and leads his men to Scylla with a bad conscience.

It has been argued that these changes may reflect not just artistic or even cultural tendencies, but profound alterations in the mental structure of those who wrote, collected and assimilated the stories. Marshall McLuhan, in his seminal work on the relationship between semiotics, media and thought structures, argued for a materialistic effect of the type of medium (the linearity of written language, the holistic nature of the moving image) on the organization of thoughts (linear or integrative, respectively) (McLuhan, 1962). Given that one of the defining features of the Axial Age is the widespread adoption of the written word, it is reasonable to consider that the transitions from orality to literacy and from lower to higher introspection are fundamentally linked (Ong, 1982). Jaynes, moreover, proposed that the transition was also accompanied by a change from a mentality dominated by inner voices, issuing god-like commands necessary to maintain social cohesion, to one where the voices were replaced by a self-aware inner dialogue. Equally interesting and controversial from a neuroscientific perspective (Kuijsten, 2006; Cavanna et al., 2007), these ideas cannot be validated or refuted solely on the basis of philological and cultural studies. However, the “soft” hypothesis, as Daniel Dennett termed it, that within the Judeo-Greco-Christian tradition at least, there exists an “arrow of time” in the cultural narrative of the Axial Age representing increased concern with *introspection* can be put to the test in a quantifiable framework (Dennett, 1986; Jaynes, 2000).

The increasing availability of large text databases has facilitated the study of the statistical regularities embedded in language, including literary subtleties such as the analysis of a novel's plot, traditionally restricted to qualitative assessments (Moretti, 2005). Here we set out to develop a consistent analytic framework to measure the degree of introspection in extant texts spanning the Axial Age. With this tool, we tested the hypothesis of a “quantum leap” in the narrative during this transitional period, more generally, the possibility of measuring a *cultural history of introspection*.

We understand meaning in language as arising from the mutual dependencies of concepts, in a holistic fashion (Quine, 1951; Cancho and Sole, 2001; Sigman and Cecchi, 2002). Hence, our challenge is not merely to count the occurrence of a given word (for example, introspection) in a historical corpus but, instead, to measure the extent to which a concept, in its distributed semantic sense (as captured, for instance, in dictionaries, and thesauri), is represented in the text. Several methods have been introduced to identify regularities and obtain a notion of semantic proximity (Lund and Burgess, 1996; Patwardhan et al., 2003; Pedersen et al., 2004; Fellbaum, 2010). One of the more widely used resources is Latent Semantic Analysis (LSA) (Deerwester et al., 1990). LSA assumes that semantically related words will necessarily co-occur in texts with coherent topics. For a concept such as introspection to convey meaning, it must evoke a large array of related concepts or “images” in the mind. Conversely, semantically related words can convey the meaning of introspection without the word itself being present in a text. This is particularly relevant for our study of classical literature, as we do not expect words representing higher-order concepts such as introspection to be present in the Bible or Homer at all. That is, the use of semantic similarity analysis allows us to measure the relatedness of a classical text to a concept with no explicit word at the time.

Our goal in the present study is to quantify the incidence of introspective topics in Axial Age literature, and uncover its cultural history. For this purpose, we decided to analyze two relatively self-consistent cultural traditions: the Judeo-Christian, anchored by the Biblical texts, and the Greco-Roman, whose foundational text is the Homeric saga, but include also the classical Greek and Latin writings up to the second century CE, encompassing among others Plato and Aristotle's extant oeuvre. Furthermore, we extended this analysis to modern times, by measuring the evolution of introspection in the Google n-grams database (Michel et al., 2011) during the twentieth century. While this database consists of an aggregate of word frequencies in the entire literary production per year, and thus it is qualitatively different from the book-based classical corpus, it allows us to explore to what extent the dynamics of introspection can be coupled to factors other than literacy.

## Materials and methods

### Texts

For the analysis of the Judeo-Christian tradition, we downloaded the Old and New Testaments, as well as St. Augustine's *Confessions* from the Project Gutenberg database (Hart, 2011). The Greco-Roman texts were downloaded from the MIT Classics archive (Stevenson, 2010), all in plain-text format (see Appendix for details). Most documents had headers and footers with commentaries and information about the source database which where cropped by hand; only the title and full text of each book was considered for this study.

We preprocessed each text in this corpora using the NLTK (Natural Language Toolkit) library (Bird et al., 2009). As a first step, we word-tokenized the text, discarding punctuation marks and symbols. The result is a list of words for each text, with repetitions. We then lemmatized the text. First, we tokenized each text into sentences, and POS-tagged (part of speech) them using the Treebank tagger supplied by NLTK. Once each word was tagged, we lemmatized each sentence using NLTK's WordNet lemmatizer. The result of preprocessing is a list of lemmatized words, each one in a new line maintaining original order, in lowercase and without any punctuation marks or symbols.

For the analysis of the modern era, we used the Google n-grams database (Michel et al., 2011). This database consists of all the words found each year on the books digitized by Google, along with their frequencies for that year. In this case no lemmatization was done, as words are isolated from the sentence where they were written. Words without the sentence are impossible to tag into their Part of Speech, impeding correct lemmatization.

### Semantic analysis

To establish a measure of semantic similarity between each pair of words, we used LSA (Deerwester et al., 1990), a high-dimensional associative model that captures similarity between words. LSA generates a linear representation of the semantic content of words based on their co-occurrence with other words in a text corpus. If the corpus is sufficiently large and diverse, the frequency of co-occurrence of words across different documents represents the extent to which the words are semantically related (Landauer and Dumais, 1997).

The input to LSA is a word-by-document occurrence matrix *X*, with each row corresponding to a unique word in the corpus (*N* total words) and each column corresponding to a document (*M* total documents). An entry *x*_*ij*_ in this matrix indicates the number of occurrences of word *i* in document *j*. Using Singular Value Decomposition (SVD), the dimensionality of this matrix is reduced to a smaller number of columns, preserving as much as possible the similarity structure between rows. Formally, using SVD, we obtain a decomposition *X*_*N* × *M*_ = *U*_*N* × *R*_ *S*_*R* × *R*_ *V*_*R* × *M*_, where *R* is the rank of matrix *X*. If we reduce dimensionality to *k* dimensions, $X_{N\times M}\approx {\tilde{X}}_{N\times M}={\tilde{U}}_{N\times k}{\tilde{S}}_{k\times k}{\tilde{V}}_{k\times M}$, where *k* < *R* and $\tilde{U},\;\tilde{S},\;\tilde{V}$ are the same as the original decomposition (*U*, *S*, *V*) cropped to *k* dimensions. By reducing dimensionality, each word is projected into a space where semantic “meaning” is just its corresponding vector. The similarity in meaning between two words can be measured by calculating the cosine between the corresponding vectors. That is, similarity between two words is computed as $W(a,\;b\text{)}=\vec{a}\cdot \vec{b}$, where the vectors are the SVD representation of the words *a* and *b*. Since the vectors are normalized, the range of possible values for the similarity measure is (−1, 1).

An important step in LSA is the generation of the word-by-document matrix from which semantic directions are extracted. We decided to use the TASA corpus, a collection of educational materials, compiled by Touchstone Applied Science Associates to develop The Educator's Word Frequency Guide. The TASA corpus consists of 37, 651 documents and 12, 190, 931 words, from a vocabulary of 94, 796 distinct words. The materials in TASA consist of general reading texts assumed to be common in the US educational system up to 1st year of college, including a wide variety of short documents taken from novels, newspaper articles, and other sources. This corpus is frequently used in LSA applications.

After generating the occurrence matrix for TASA, SVD was executed obtaining the decomposition. As the LSA method proposes, the SVD matrix may be cropped—reducing dimensionality—conserving the range of the original matrix. The choice of dimensionality is an important factor for success in measuring distance of concepts. Landauer and Dumais studied the effect of the number of dimensions retained in LSA and word-meaning similarities (Landauer and Dumais, 1997). The maximum performance was obtained retaining around 300 dimensions, the number we used in this study.

For the analysis of the Judeo-Christian and Greco-Roman corpora, texts were lemmatized. Thus, the TASA corpus was also preprocessed in the exact same way, including lemmatization. This processing resulted in a vocabulary of 78, 633 distinct words, 17% smaller than the un-lemmatized TASA corpus. For the analysis of the Google n-grams, the complete un-lemmatized TASA corpus was used.

## Results

### Judeo-Christian texts

Due to the relative uncertainty of dating its different books, we collapsed the Old Testament into a single document, to be compared against the New Testament. Merely for the purpose of graphical depiction, we approximated the average dating of the Old Testament to the fourth century BCE given the wide spread of possible dating from the oldest books [second millennium to fifth century BCE, although in their written form they are more recent (Schniedewind, 2004)] to the newest ones (Maccabees, second-first century BCE) (Coogan, 1998). The dating of the New Testament is comparably much more confined being certainly constrained to the second century CE.

In order to compare these results with a more modern text of the same tradition, we performed the same analysis on the *Confessions*, written by St. Augustine (or Augustine of Hippo in the Protestant nomenclature) around 398 CE. This book, an auto-biography, is considered a fulcrum between the classic and modern conceptions of the self, infused with Augustine's idea of free will and self-determination (Brown, 1967; Caputo and Scanlon, 2005).

**Figure 1:**
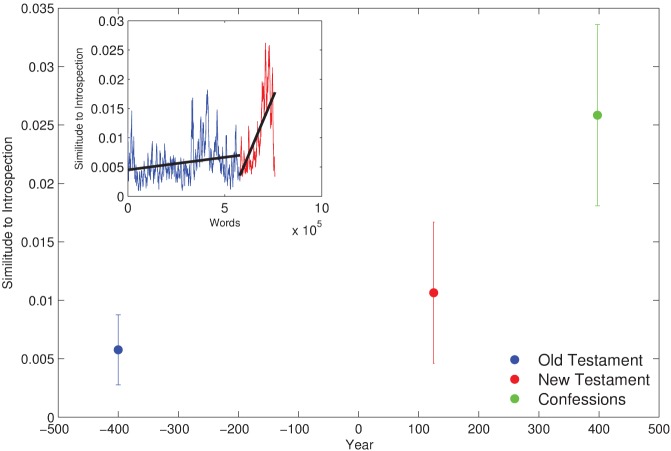
**Introspection in the cultural record of the Judeo-Christian tradition.** The New Testament as a single document shows a significant increase over the Old Testament, while the writings of St. Augustine of Hippo are even more introspective. Inset: regardless of the actual dating, both the Old and New Testaments show a marked structure along the canonical organization of the books, and a significant positive increase in introspection.

For each book (Old Testament, New Testament, and Confessions), we calculated LSA semantic similarity *W*_*i*_ between each single word and the word “introspection,” resulting in a series with values between −1 (semantically dissimilar to introspection) and 1 (semantically similar to introspection). We binarized these values to boost the weight of highly introspective words, using an arbitrary threshold of 0.15, so that for each word *i* in the text we assign $S_{i}=1\text{ if }W_{i}=\vec{w}\cdot \vec{I}>0.15,\;S_{i}=0$ otherwise, where ${\vec{w}}_{i},\;\vec{I}$ are, respectively, the LSA representation of word *i* and of *introspection*. We finally computed a moving average of this series with a window of 500 words, representing an estimate of the local similarity to introspection of each fragment of the text. This series is shown explicitly for the Old and New Testaments in the inset of Figure 1, and represented by its mean and standard deviation for the three books in the main plot.

The Old and New Testament are significantly different in terms of their semantic similarity to introspection (*t*-test, *p* < 10^−12^), in agreement with the transition hypothesis. The *Confessions* further shows a significant increase in introspection from the New Testament (*t*-test, *p* < 10^−12^), consistent with its placement in the post-transition era, and with its qualitative scholarly assessment. As we mentioned above, we can only rely on broad, approximate dates for the compilation and writing of the books that conform the Old Testament, in particular for the oldest ones. The final *order* of the books in the canonical format we know today is as much a reflection of their temporal precedence as of multiple editorial interventions (Scholes and Kellogg, 1966). We therefore found highly suggestive that our semantic analysis term-by-term along the textual linearity of both Old and New Testaments also shows a statistically significant trend toward increasing introspection. This is represented in the inset of Figure 1, where the moving average follows the textual precedence order of the Old (blue) and New (red) Testaments. Linear regression shows a significant positive slope (for Old Testament, *m* = 4 × 10^−9^, *p* < 10^−12^; for New Testament, *m* = 8 × 10^−8^, *p* < 10^−12^). While this trend is markedly positive on the latter, it is still significant in the former. A detailed analysis of the structure of the New Testament is beyond the scope of this paper; let us note, however, that the marked increase in the similarity to introspection corresponds approximately to the Pauline epistles.

### Greco-Roman texts

As it is the case with the oldest Judeo-Christian texts, it is difficult to precisely date the Homeric saga. Recent studies have dated with certain confidence the Trojan War and events related to the Odyssey around 1200 BCE (Baikouzis and Magnasco, 2008), while the compilation into a coherent corpus probably dates between 900 and 800 BCE, and the final composition in the newly introduced Greek alphabet between 700 and 650 BCE (Ong, 1982). While Jaynes dates the two epics roughly a century apart (Jaynes, 2000), we only assume some form of temporal precedence affected by the introspective transition hypothesis.

**Figure 2:**
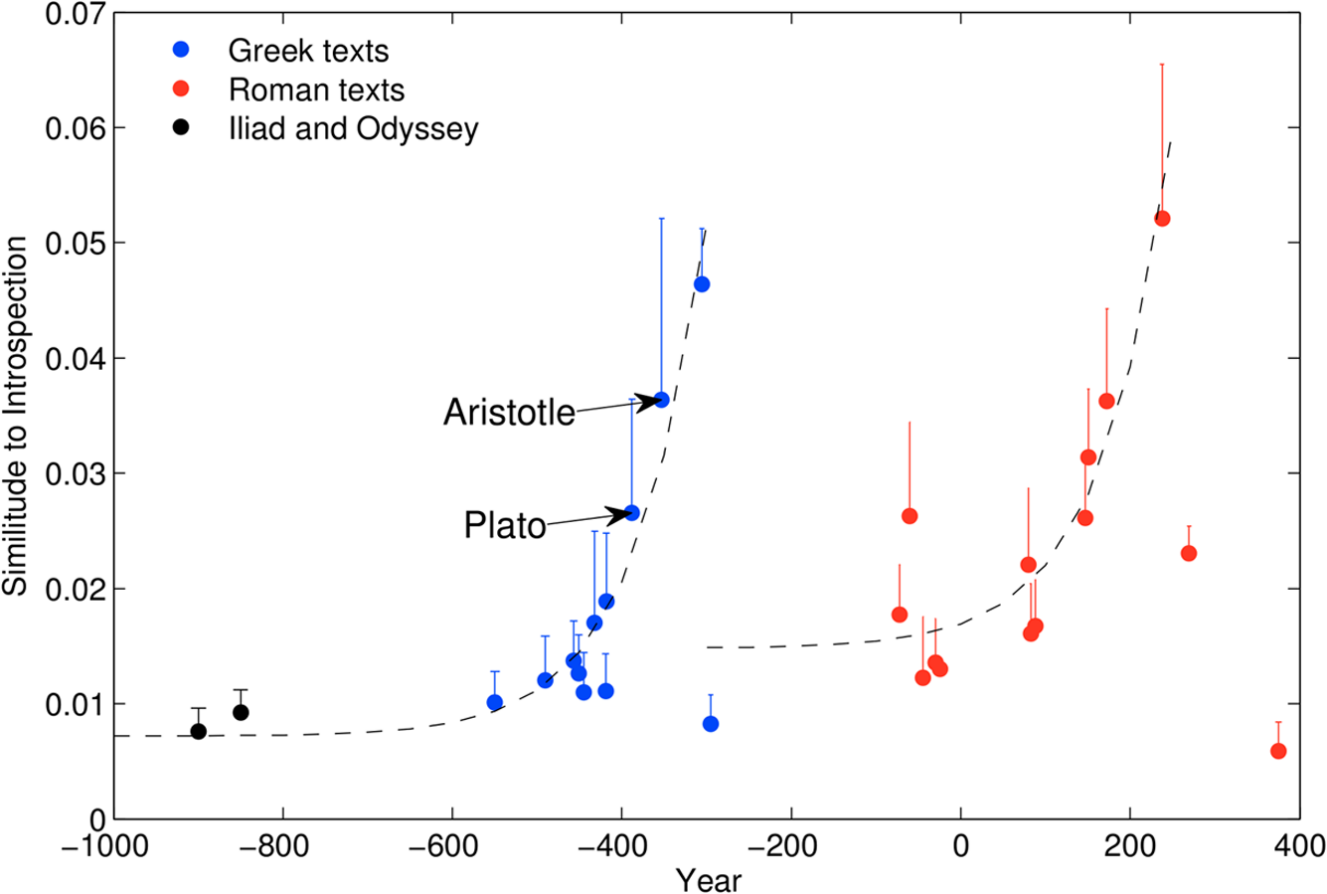
**Introspection in the cultural record of the Greco-Roman tradition.** Dotted lines represent exponential fits for each of the traditions.

Similarity to introspection for all Greco-Roman books was performed with the same approach used for the Judeo-Christian texts. The full figure shows the same analysis extended to the database of extant Greco-Roman texts (Figure 2), for which dating is significantly firmer than for the Homeric saga. As suggested by Jaynes, and in line with the broader Axial Age idea, we observe that in the period up to 400–300 BCE, the literary record displays a dramatic divergence toward higher introspective value (fit to an exponential function: *R*^2^ = 0.92, *p* < 10^−12^, see Appendix for details).

Following approximately the end of Greece's Golden Era, however, introspective content in the literary record displays an abrupt fall, followed in its turn by a second rise, mimicking the previous transition, essentially with Roman writings. The Roman rise is less abrupt than the Greek one, but nevertheless clearly significant (*R*^2^ = 0.85, *p* < 10^−12^).

### Modern cultural record

The finding of two “transitions” in the Greco-Roman tradition implies a possible link between introspection and societal upheaval. In order to test this hypothesis, we decided to investigate the semantic relatedness of introspection in the modern cultural record compiled in the *Google n-grams* database (Michel et al., 2011). From all words included in this database, we filtered those included in the LSA dictionary—that is, all different words included in TASA. As mentioned in the Materials and Methods section, we used the un-lemmatized TASA-trained LSA, as words in the n-grams database lack their sentence context.

**Figure 3:**
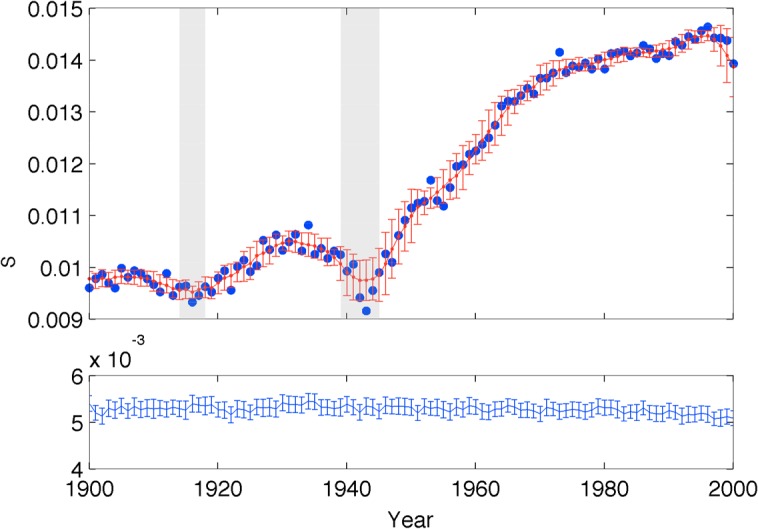
**Evolution of introspection in the modern cultural record. Top**: the blue circles correspond to semantic relatedness to introspection, and the red trace to the mean and standard deviation of these values on a 6-year running window (i.e., running average and nosie). The gray shades correspond to the two World Wars, and the red vertical dashes the standard deviation of the null hypothesis. **Bottom**: mean and standard deviation of the null hypothesis corresponding to randomized word frequencies.

For each year, we calculated the semantic relatedness of introspection as *f*(*y*),

(1) $$f(y)=\sum_{i=1}^{N(y)}\frac{W_{w_{i}(y)}\times A_{w_{i}(y)}}{T(y)}$$

where *N*(*y*) is the number of different words during year *y* (intersected with those available in TASA), *W*_*w*_*i*_(*y*)_ is the introspective weight of word *w*_*i*_(*y*) as measured by its LSA distance to “introspection,” *A*_*w*_*i*_(*y*)_ is the number of occurrences of word *w*_*i*_(*y*) during year *y* and *T*(*y*) = ∑_*j* = 1_^*N*(*y*)^ *A*_*w*_*j*_(*y*)_ is the total words during year *y*. In order to assess the statistical significance of yearly changes, we computed a local measure of noise as the standard deviation of the mean of *f*(*y*) in a 6-year window centered in *y*. The results are shown in Figure 3-top, where the twentieth century CE was analyzed.

To verify the significance of the measurement for each year, we computed the expected similarity value for the null hypothesis that the results obtained can be simply explained by the distribution of similarity to introspection of the word ensemble for the year, regardless of the frequency of occurrence. For that, we generated 100 surrogate trials with randomized (uniform) frequencies for each word every year *y*, and calculated *f*_null_(*y*). In Figure 3-bottom, we show the average and standard deviation of these surrogates. We confirmed the significance of the introspective value as each year surrogates are very distant to introspection compared to the n-gram values (all *t*-test's with *p* < 10^−8^).

We observe a marked effect of WWI and WWII, which coincide with significant troughs, or at least punctuate the inter-war peak and a sustained increasing arc following WWII. While we can only speculate about what the significance of this arc means, the correlation between war and drops in introspection has obvious implications and is also consistent with our observation of the Greco-Roman cultural record.

It is possible that the growth of introspection observed in different cultural records merely reflects a cumulative effect of culture and knowledge. Similarly, it can be argued that the between-wars drop of introspective content in the cultural record may simply reflect a global trend that can be manifested by a broad number of concepts, not only introspection.

To investigate the specificity of this effect to introspective content, we repeated the same analysis on a list of 200 concepts deemed “fundamental” across 87 Indo-European languages (Pagel et al., 2007). A visual inspection clearly reveals that all the main effects reported here are specific to introspection, and do not replicate across these 200 other concepts (see Appendix for details and figures). By specificity we do not mean that this is the sole concept to show this dynamics, we simply argue that it is not a general trend observed in concepts which show less cultural dependence. First, these words did not show a systematic growth as compared, for example, to the growth of introspection in the New Testament. In the modern cultural records, control words did not show any systematic trend revealing, as we observe for introspection, a rise and fall between wars and a consistent progression after the second world war. To quantify this observation we performed two independent statistical tests. We measured the quadratic dependence of each word during the period 1914–1939 (from the beginning of the first to the beginning of the second world war) and the linear dependence in the 1945–2000 period (from the end of the second war to the end of the century). The quadratic term for introspection (1914–1939) was negative, reflecting a convex dependence (rise and then fall) with a significance of *p* < 0.01, considering as the null-hypothesis the distribution of the quadratic terms obtained from all control words. Similarly, the post-war growth was testified by a positive linear correlation which was also within the one percentile of all the control words. The growth of introspection in the New Testament was also significant within the distribution of control words (*p* < 0.01).

## Discussion

In light of the results obtained analyzing the historical literary record of the two root traditions of the Western civilization, a number of questions are left open. From a neuroscientific perspective the most prominent is, whence comes the transition? Numerous scholars have pointed to the consolidation of alphabetic writing during the Axial Age as one of the main causes of a change in the cultural dynamics of introspection (Ong, 1982; Jaynes, 2000). The Greek alphabet, in particular, developed around 700 BCE, had finally been internalized in the culture by Plato's time. Notably, however, Socrates, in Plato's *Phaedrus*, excoriates it on the basis of its corruption of the faculty of memory (Stevenson, 2010). It is precisely this effect on memorization that has been regarded as introducing a dramatic change in mental life: by easing the onerous task of memorizing the narrative that defines a culture, it allows the mind to wander with new freedom, and releases it from the constant anchor of “internalized external voices” (Ong, 1982). As mentioned in the Introduction, McLuhan similarly relates the linearity and constrained structure of the written text to the development of rational thinking and the abandonment of “magical” or unconstrained form of thought, typical of orality (McLuhan, 1962).

There is evidence that literacy is associated with functional as well as anatomical changes in the brain of literate as opposed to illiterate adults, even when literacy is acquired later in life (Petersson et al., 2007; Carreiras et al., 2009; Dehaene et al., 2010). While these observations do not have a direct bearing on the possible link between literacy and introspection, recent findings indicate an association between brain symmetry and commanding hallucinations and psychosis (Laval et al., 1998; Olin, 1999; Corballis, 2008). It is therefore reasonable to hypothesize that the strong lateralization that accompanies literacy can influence the mental processes required for increased introspection. Moreover, introspection, at least in the restricted sense of the meta-cognitive ability to judge your own performance in a task, also has measurable physiological correlates (Kiani and Shadlen, 2009; Fleming et al., 2010), and therefore cannot be dismissed as an ephemeral cultural by-product.

A typical argument leveled against the hypothesis of a historical evolution of introspection is that even in the relatively backward civilization of the Homeric epics and Ancient Israel, people were organized in complex social structures that required equally complex behaviors including planning, assessment and a form of “theory of mind,” among other cognitive features inconsistent with a non-introspective mind. While it would indeed be non-sensical to assume that pre-Axial Age humans were mere automata, incapable of any form of reflexion, a growing body of research suggests that goal-oriented behavior in individuals is highly determined by social imposition, and can occur largely outside awareness (Custers and Aarts, 2010).

Our analysis of the Greco-Roman and modern cultural records suggests that the dynamics of introspection are heavily determined by, or at least correlated with, the overall state of the society and liable to dramatic changes over short periods of time. We cannot consider this finding as a refutation of the hypothesis of a link between the orality-to-literacy transition and increased introspection, as a relatively thriving societal context may be a necessary pre-requisite but not a sufficient cause. Moreover, we need to consider that the relationship between outward cultural manifestations and the mental makeup of individuals in a culture is highly complex and devoid of a clear causal directionality. In fact, the rapid changes we observe around the World Wars may be interpreted precisely as indicating that cultural productions have a dynamics of their own, not reducible to a simple reaction to dramatic “external” factors.

To the extent that the texts we studied represent Axial Age civilizations, our results provide a framework on which hypothesized cultural changes affecting cognitive structures can be quantifiably measured. By the same token, our analytic approach can be extended to characterize other similarly high-level cognitive features in the cultural record as a whole, literary styles (Moretti, 2011) and psychiatric dysfunction (McKenna and Oh, 2005; Mota et al., 2012).

### Conflict of interest statement

The authors declare that the research was conducted in the absence of any commercial or financial relationships that could be construed as a potential conflict of interest.

## Figures and Tables

**Figure A1:**
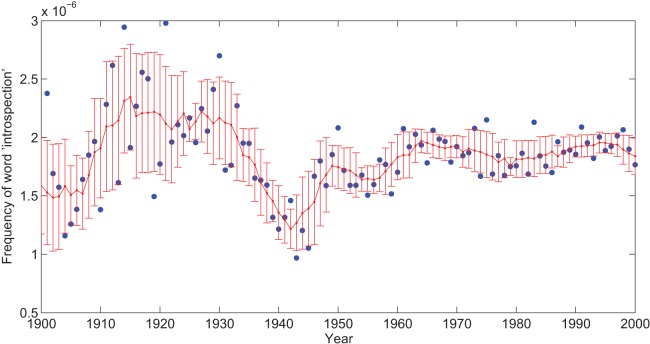
**Frequency of the term “instrospection” on Google n–grams in the twentieth century.** The blue points correspond to frequency of the word introspection, and the red bars indicate the mean and standard deviation of the null hypothesis.

**Figure A2:**
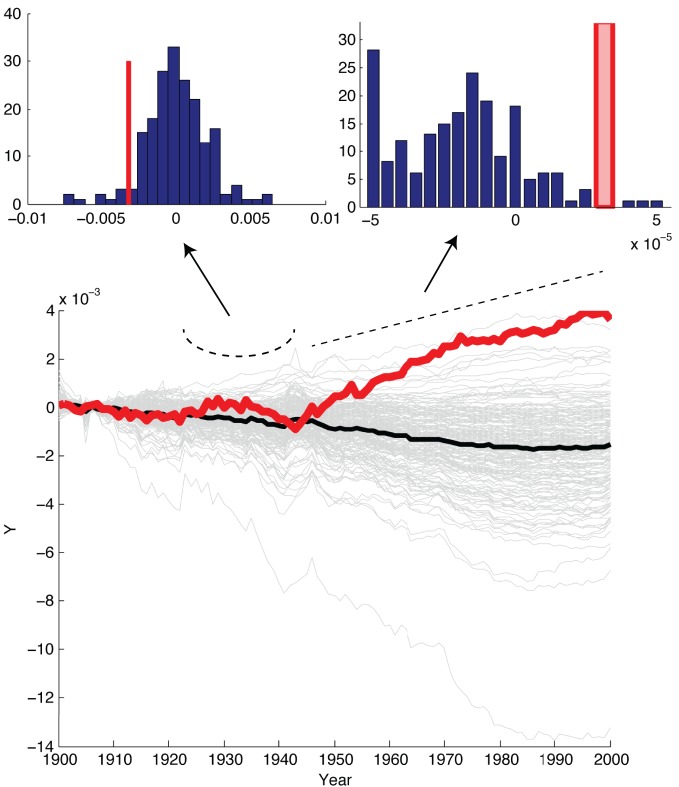
**LSA distance to “introspection” and 200 control words in the Google n–grams.** The red line corresponds to introspection, and the gray lines to each of the control concepts. The dark black line is the mean of all concept words. To facilitate a visual comparison, each curve has been normalized to its mean in the first 10 years (1900–1910). A quadratic term was fit to the period 1914–1939 and a linear term to the period 1945–2000. The inset bar plots show, in blue, the distribution of terms for each of the control words. In red, it shows the corresponding term for introspection.

**Figure A3:**
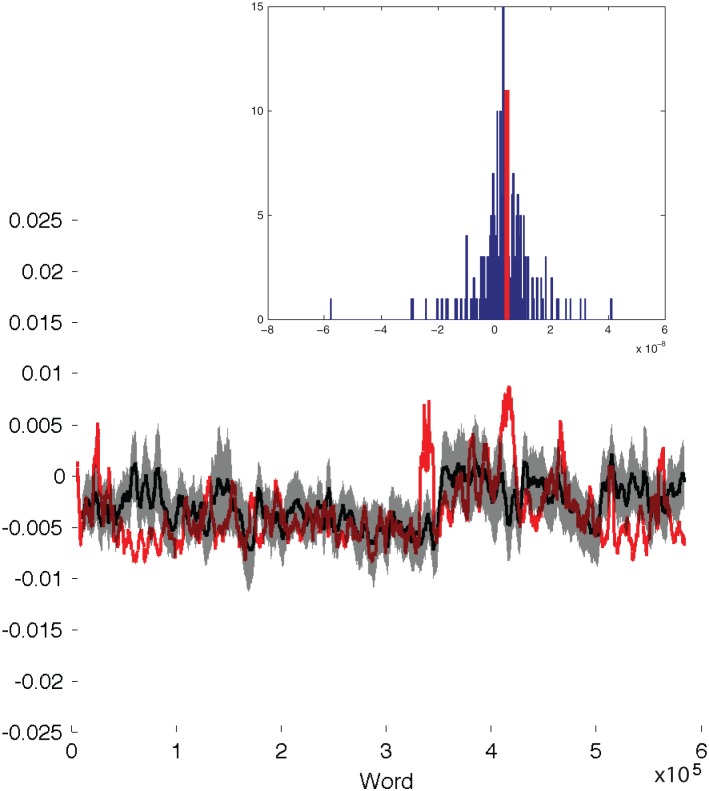
**LSA distance to control words in the Old Testament.** Black curve shows the mean distance for the control words, with the gray shadow indicating standard deviation. The red curve corresponds to introspection.

**Figure A4:**
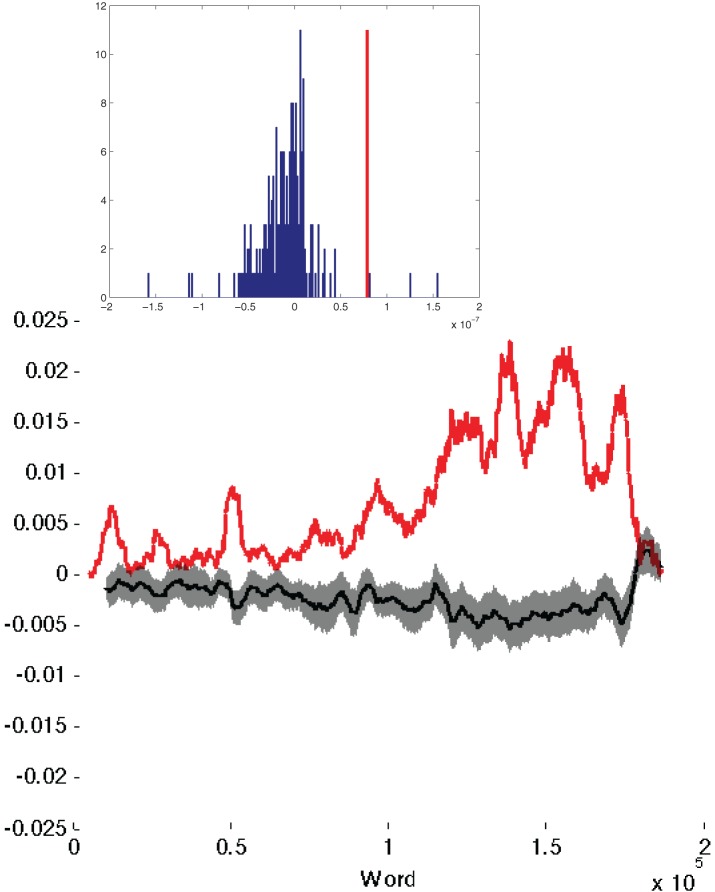
**LSA distance to control words in the New Testament.** Black curve shows the mean distance for the control words, with the gray shadow indicating standard deviation. The red curve corresponds to introspection.

## Appendix

### Judeo–Christian texts

For the analysis of the Judeo–Christian texts, we used two documents: (1) the Bible (Old and New Testaments); and (2) the Confessions of St. Augustine.

There are many versions of the Bible available. We used the King James version available online at the Project Gutenberg (http://www.gutenberg.org/ebooks/10). We downloaded the *Plain Text UTF–8* version (http://www.gutenberg.org/cache/epub/10/pg10.txt), and cropped header and footer containing information about Project Gutenberg.

The Confessions by St. Augustine was also downloaded from Project Gutenberg: *The Confessions of St. Augustine by Bishop of Hippo Saint Augustine* (http://www.gutenberg.org/cache/epub/3296/pg3296.txt).

### Greco–Roman texts

We downloaded all Greco–Romans texts from the MIT Internet Classic Texts site: http://classics.mit.edu/, in their English translations. We grouped the texts by author, that is, all documents from each author were concatenated in the same file. In Table A1 we enumerate the authors and dates corresponding to the texts.

**Table A1:** **Greco–Roman authors**.

**Author**	**Orig. Lang.**	**Date**
Aeschines	Greek	390–314 B.C.E.
Aeschylus	Greek	525–456 B.C.E.
Aesop	Greek	6th century B.C.E.
Andocides	Greek	440–391 B.C.E.
Antiphon	Greek	480–411 B.C.E.
Apollodorus	Greek	140 B.C.E.
Apollonius	Greek	ca. 295 B.C.E.
Apuleius	Latin	124 A.C.E.–ca. 170 A.C.E.
Aristophanes	Greek	450–388 B.C.E.
Aristotle	Greek	384–322 B.C.E.
Marcus Aurelius	Greek	121–180 A.C.E.
Augustus	Latin	63 B.C.E.–14 A.C.E.
Bacchylides	Greek	5th century B.C.E.
Julius Caesar	Latin	100–44 B.C.E.
Cicero	Latin	106–43 B.C.E.
Demades	Greek	380–319 B.C.E.
Demosthenes	Greek	384–322 B.C.E.
Dinarchus	Greek	360–292 B.C.E.
Diodorus	Greek	1st century B.C.E.
Epictetus	Greek	55–105 A.C.E.
Epicurus	Greek	341–270 B.C.E.
Euclid	Greek	ca. 300 B.C.E.
Euripides	Greek	484–406 B.C.E.
Galen	Greek	129–216 A.C.E.
Herodotus	Greek	484–430 B.C.E.
Hesiod	Greek	700 B.C.E.
Hippocrates	Greek	460–377 B.C.E.
Hirtius	Latin	90–43 B.C.E.
Homer	Greek	800 B.C.E.
Horace	Latin	65–8 B.C.E.
Hyperides	Greek	390–322 B.C.E.
Isaeus	Greek	420–350 B.C.E.
Isocrates	Greek	436–338 B.C.E.
Josephus	Greek	37–100 A.C.E.
Livy	Latin	59 B.C.E.–17 A.C.E.
Lucan	Latin	39–65 A.C.E.
Lucretius	Latin	1st century B.C.E.
Lycurgus	Greek	390–324 B.C.E.
Lysias	Greek	445–380 B.C.E.
Ovid	Latin	43 B.C.E.–17 A.C.E.
Pausanias	Greek	143–176 A.C.E.
Pindar	Greek	518–438 B.C.E.
Plato	Greek	428–348 B.C.E.
Plotinus	Greek	205–270 A.C.E.
Plutarch	Greek	46–119 A.C.E.
Porphyry	Greek	234–305 A.C.E.
Quintus	Greek	ca. 375 A.C.E.
Sophocles	Greek	496–406 B.C.E.
Strabo	Greek	64 B.C.E.–23 A.C.E.
Tacitus	Latin	56–120 A.C.E.
Thucydides	Greek	460–404 B.C.E.
Virgil	Latin	70–19 B.C.E.
Xenophon	Greek	431–349 B.C.E.

### Google n–grams

To study the amount of introspection in the modern cultural record we used the Google n–grams database. This database consists of “a corpus of 5,195,769 digitized books containing ~4% of all books ever published” (Michel et al., 2011). We downloaded the raw data for 1-grams from http://books.google.com/ngrams/datasets, version 20090715.

### Frequency vs. LSA distance

To verify the need for a semantic distance metric in our analysis of introspection on the Google n–grams, we compared against a simple search for the word “introspection” along the twentieth century. Figure A1 shows the frequency with which the word appears each year. While it is possible to observe the decline between the Great Depression and the end of WWII, the dramatic increase we observed in the post–war cannot be observed here.

### Control concepts

As mentioned in the main text, to investigate the specificity of our results to the concept of introspection, we repeated our analyzes and performed some statistical tests on a list of 200 concepts deemed “fundamental” across 87 Indo–European languages (Pagel et al., 2007).

Figure A2 shows the LSA distance to “introspection” and the set of 200 control concepts for the Google n–grams. By mere visual inspection we can observe a dramatic shift in the post–war period, when we compare the evolution of introspection against the semantic distance to other control words, and in particular against the mean distance. Moreover, control words did not show a systematic trend in relation to the World Wars, as we have shown for the case of introspection.

We perform two statistical tests to quantify these effects. First, we fit a quadratic term to the period 1914–1939. In the case of introspection, this term was negative, reflecting the observed rise and then fall of introspection in the period. The first inset in Figure A2 shows the distribution of quadratic terms for each of the 200 control words, and in red the value of this term in the case of introspection. Taking the distribution for control words as our null hypothesis, we observe that the value of the introspection term is significantly outside this distribution (*p* < 0.01).

Second, we fit a linear term to the period 1945–2000, the second half of the century after the wars. Again, the distribution of these terms for the control words is shown in blue in the second inset of Figure A2, and the corresponding term for introspection is shown in red. The value of the introspection term is significantly outside the distribution (*p* < 0.01).

We performed a similar control for the case of the Bible. Figure A3 compares the LSA distance of each term in the Old Testament against introspection (red curve) and all control words (gray lines). The inset in the figure shows the distribution of linear fit coefficients to each curve. In this case, the coefficient for the Old Testament is not significantly outside the distribution for control terms.

Figure A4 compares introspection and control words in the New Testament. The growth of introspection in the New Testament is significantly outside the distribution of control words at the *p* < 0.01 level.
